# Genome-wide identification, phylogenetic and expression pattern analysis of HSF family genes in the *Rye* (*Secale cereale* L.)

**DOI:** 10.1186/s12870-023-04418-1

**Published:** 2023-09-20

**Authors:** Yanyan Ren, Rui Ma, Muhua Xie, Yue Fan, Liang Feng, Long Chen, Hao Yang, Xiaobao Wei, Xintong Wang, Kouhan Liu, Peng Cheng, Baotong Wang

**Affiliations:** 1 State Key Laboratory of Crop Stress Biology for Arid Areas, College of Plant Protection, Northwest A&F University, Yangling, Shaanxi 712100 People’s Republic of China; 2 College of Food Science and Engineering, Xinjiang Institute of Technology, Aksu, 843100 People’s Republic of China; 3 Chengdu Institute of Food Inspection, Chengdu, 610000 People’s Republic of China; 4 Tianfu New Area General Aviation Profession Academy, Meishan, 620564 China; 5 Agricultural Service Center of Langde Town of Leishan County, Qiandongnan Miao and Dong Autonomous Prefecture, 556019 China; 6 Guizhou Provincial Center For Disease Control And Prevention, Guiyang, 550025 People’s Republic of China

**Keywords:** *Rye*, *HSF* gene family, Hormone, Abiotic stress

## Abstract

**Background:**

Heat shock factor (HSF), a typical class of transcription factors in plants, has played an essential role in plant growth and developmental stages, signal transduction, and response to biotic and abiotic stresses. The *HSF* genes families has been identified and characterized in many species through leveraging whole genome sequencing (WGS). However, the identification and systematic analysis of *HSF* family genes in *Rye* is limited.

**Results:**

In this study, 31 *HSF* genes were identified in *Rye*, which were unevenly distributed on seven chromosomes. Based on the homology of *A. thaliana*, we analyzed the number of conserved domains and gene structures of *ScHSF* genes that were classified into seven subfamilies. To better understand the developmental mechanisms of *ScHSF* family during evolution, we selected one monocotyledon (*Arabidopsis thaliana*) and five (*Triticum aestivum* L., *Hordeum vulgare* L*.*, *Oryza sativa* L*.*, *Zea mays* L., and *Aegilops tauschii* Coss.) specific representative dicotyledons associated with *Rye* for comparative homology mapping. The results showed that fragment replication events modulated the expansion of the *ScHSF* genes family. In addition, interactions between ScHSF proteins and promoters containing hormone- and stress-responsive cis-acting elements suggest that the regulation of *ScHSF* expression was complex. A total of 15 representative genes were targeted from seven subfamilies to characterize their gene expression responses in different tissues, fruit developmental stages, three hormones, and six different abiotic stresses.

**Conclusions:**

This study demonstrated that *ScHSF* genes, especially *ScHSF1* and *ScHSF3*, played a key role in *Rye* development and its response to various hormones and abiotic stresses. These results provided new insights into the evolution of *HSF* genes in *Rye*, which could help the success of molecular breeding in *Rye*.

**Supplementary Information:**

The online version contains supplementary material available at 10.1186/s12870-023-04418-1.

## Background

*Rye* (Secale cereale, 2n = 2x = 14, RR) belongs to the genus Secale in the Triticeae tribe of Poaceae, the grass family [1]. It is a good source of carbohydrates and a small amount of protein, potassium, B vitamins, lignans, ferulic acid, alkylpolysechenol, and prebiotics. *Rye* is mainly used for bread production and high-quality animal feed and pasture [2, 3]. In addition, *Rye* exhibits strong tolerance to abiotic stress, such as cold tolerance, drought resistance and soil impoverishment resistance, as well as biotic stress, including resistance to fungi and other pathogens [4, 5]. Recently, numerous studies have been devoted to studying *Rye* genome, which has laid the foundation for the promotion of breeding and targeted gene editing in *Rye* [6, 7].

Transcription factors (TFs), are DNA-binding proteins that specifically interact with cis-acting elements of the eukaryotic proteins [8]. As an essential class of regulators, transcription factors are involved in almost all biological processes in plants. When plants are subjected to biotic and abiotic stresses, transcription factors bind to the specific promoter regions of genes to activate or inhibit transcription of downstream target genes for defensive responses [9, 10]. Previous studies have demonstrated that heat shock transcription factors (HSFs) can regulate plant heat stress responses [11]. *HSFs* contain five basic functional and structural domains: N-terminal DNA binding domain (DBD), oligomerization structural domain (OD), nuclear localization signaling domain (NLS), nuclear export signaling domain (NES), and C-terminal transcriptional activation domain (CAD) [12, 13]. The oligomeric domain consists of two hydrophobic heptapeptide repeats, abbreviated as HR-A and HR-B. Heat shock transcription factors are divided into three subfamilies, A, B, and C, depending on the different number of amino acids inserted in HR-A and HR-B [13–15]. HSFA subfamily inserts a long amino acid chain between HR-A and HR-B, with an aromatic-rich hydrophobic acidic amino acid tail (AHA) at the C-terminus. AHA is essential for transcriptional activation function. On the other hand, the HSFB subfamily has no amino acid sequence between HR-A and HR-B, while members of HSFC subfamily have a short amino acid sequence inserted between HR-A and HR-B. Therefore, HSFA has a transcriptional activation function, while the opposite holds true for HSFB and HSFC. Under normal conditions, HSFs transcription factors are present as monomers in cytoplasm of plants and are inactive. When plants are exposed to abiotic stresses (e.g., heat stress), HSFs are active through OD binding to DNA-binding structural domains and AHA structural domains to form heat-kill transcription factor trimers. DBD structural domain will be recognized and bind to heat stress elements (HSEs) of the heat-kill protein promoter, which activates transcription of the heat-killed protein *Hsp* genes and enable plants to exhibit resistance to abiotic stresses [16–19].

Following the mining of genomic data, an increasing number of *HSF* gene families of plants such as *A. thaliana* [18], *Populus* L*.* [20], *T. aestivum* [21], *Beta. vulgaris* L. [22], and *Gossypium* spp. [23], *O. sativa* [24], *H. vulgare* [25]. and *Z. mays* [26] have been identified. Scharf first identified transcription factors *HSFs* associated with heat stress response elements by exposing cell cultures of tomatoes to heat stress. Furthermore, studies on *HSFs* have been carried out in a variety of plants [27]. In *A. thaliana*, overexpression of *AtHSFB4* (*AT1G46264.1*) gene resulted in a shortened root length. Overexpression of *AtHSFA2* improves plant salt tolerance, and promotes callus growth [28]. Overexpression of *GmHSFA1* in soybean, and overexpression of *SlHSFA1* in tomato enhances heat tolerance of transgenic plants [29, 30]. In addition, numerous studies have revealed that *HSF* family has a crucial regulatory function in various physiological aspects of the growth and development in other plants, such as stress response and plant phase transition. Currently, a number of *HSF* genes have been isolated and identified in different plants, which are still poorly understood in *Rye* [11, 31, 32].

In this study, based on the newly published *Rye* genome [7], we have identified 31 *HSF* genes and compared their gene structure, motif composition, chromosomal location, and gene duplication. To further study the developmental mechanisms among species, the *HSF* genes in *Rye* were compared with closely related genera to analyze the evolutionary distance and relationships. Finally, the expression of *HSF* genes (under hormones and stress treatment) was analyzed by qRT-PCR, it was found that there was a differential expression pattern of *HSF* genes in different tissues, which initially confirmed the biological function of *HSF* genes in *Rye*. This study provided a comprehensive analysis of the *HSF* gene family in *Rye*, which provided valuable information for screening important *HSF* genes in *Rye* under different development stages and stress treatment, and provided a theoretical basis for functional analysis of *HSF* gene family in other species.

## Result

### Identification of the *HSF* gene in *Rye*

A total of 31 *ScHSF* genes were identified according to different chromosomes and named between *ScHSF1* and *ScHSF31*. The basic characteristics of *ScHSF*, such as molecular weight (MW), isoelectric point (pI), coding sequence length (CDS), and subcellular localization (http://cello.life.nctu.edu.tw/) were analyzed.

Among the 31 ScHSF proteins, ScHSF20 protein had the least amino acids (226), while ScHSF23 protein had the most amino acids (520). The molecular weight of the protein ranged from 24.64 kDa (ScHSF20) to 57.32 kDa (ScHSF23), and the pI ranged from 4.80 (ScHSF29) to 10.24 (ScHSF20), with an average of 6.14. All ScHSF proteins contained HSF_DNA-bind domains. Subcellular localization results showed that all ScHSF proteins were located in the nucleus, while four were located in the peroxisome, three were located in the cytoplasm, and one (ScHSF13) was located in the extracellular. The number of *HSF* genes in *Rye* (31) was higher than that in *A. tauschii* Coss (19), *A. thaliana* (21), *Z. mays* (25), and *O. sativa* (26) but lower than that in *H. vulgare* (32) and *T. aestivum* (82) (Additional file 1: Table S1).

### Multiple sequence alignment, phylogenetic analysis, and classification of ScHSF proteins

To determine the phylogenetic relationships among *Rye* HSF proteins, a phylogenetic tree of *Rye* (31 ScHSFs) and *Arabidopsis* (22 AtHSFs) was constructed using MEGA 7.0 software. The 31 ScHSF proteins were divided into seven branches (groups 1–7) in the phylogenetic tree according to the previously proposed Cenci and Rouard classification method and topology [33]. Consensus exists with the taxa of HSF proteins in *Arabidopsis*, indicating that these *HSF* genes remained stable during the evolutionary process.

**Fig. 1:**
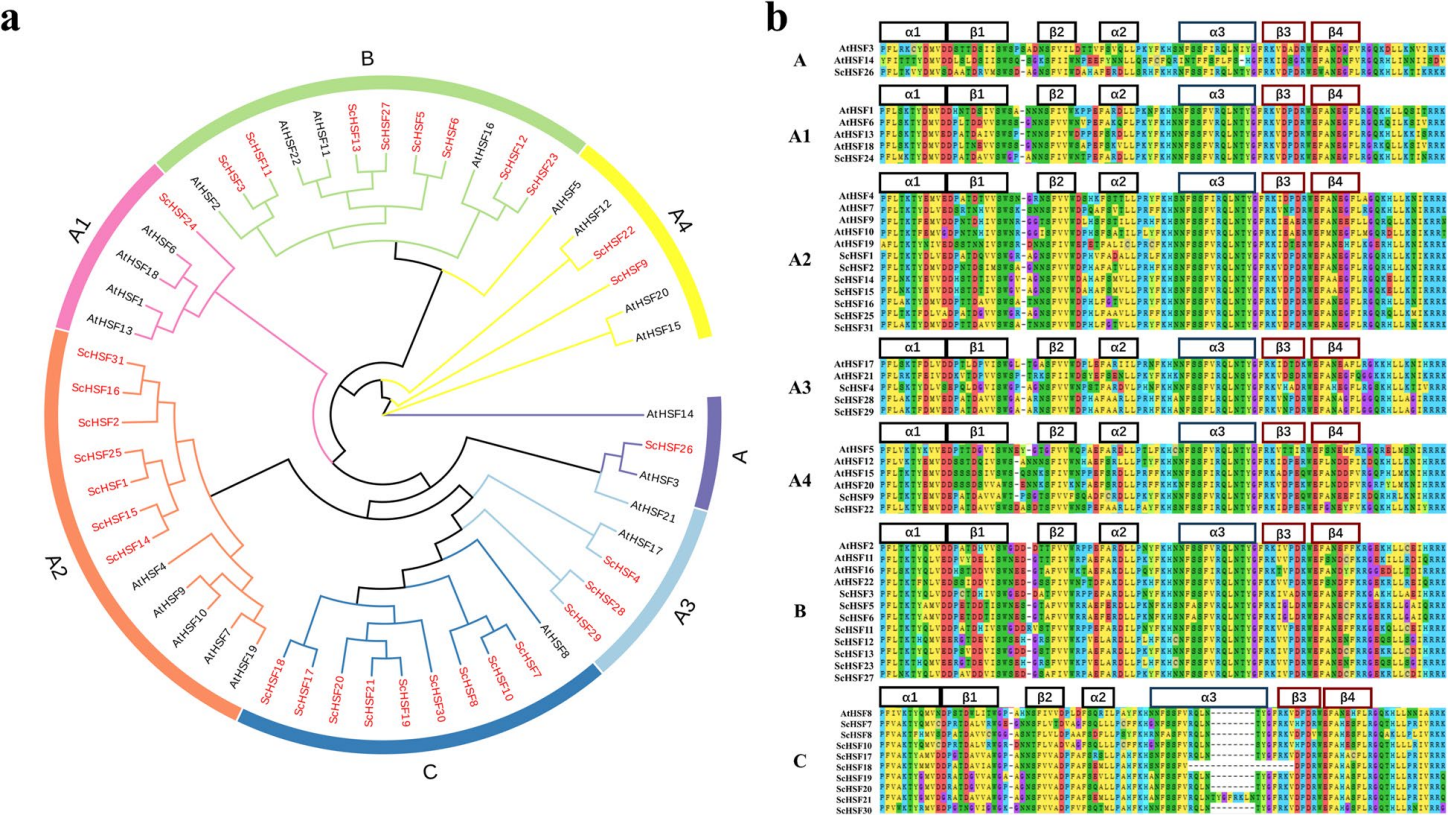
The evolutionary relationship and sequence alignment of the rye HSF proteins. **a** A phylogenetic tree of *Rye* and *Arabidopsis thaliana* HSF proteins showing that HSF proteins were divided into seven subfamilies. *Rye* proteins were denoted in red, whereas *A. thaliana* proteins were indicated in black. **b** Multiple sequence alignment of the DBD domains among the seven subfamilies of the ScHSF protein family

Among seven subfamilies, subfamily C had the largest number of members (9 ScHSFs), and subfamilies A and A1 had the fewest members (only 1 ScHSF). All members were usually concentrated in the three subfamilies A2, B and C. Comparison with the phylogenetic tree of *Rye* revealed that some of the ScHSFs clustered closely with AtHSFs (bootstrap support ≥ 70), suggesting that these proteins in *Rye* and *A.thaliana* might be homologous and have similar biological functions (Fig. 1a; Additional files 1 and 2: Table S1 and Fig. S1).

Previous studies have shown that all *HSF* proteins contain DBD-conserved domain and are highly conserved. The conserved domain contains three α-helical bundles (α1-α3) and four reverse parallel β-folds (β1-β4), consisting of about 100 amino acid residues (Fig. 1b). However, there are varying degrees of insertions or deletions in these proteins. For example, in subfamily C, *ScHSF21* had an 8 amino acid sequence inserted between α3 and β3, and *ScHSF18* had an 11 amino acid sequence missing between α3 and β3. Overall, DBD domains in *Arabidopsis* and *Rye* were highly conserved, indicating that DBD structural domains were established early in plants.

### Conserved motifs and gene structure analysis of *ScHSF* genes

The structural and taxonomic diversity of HSF genes in *Rye* were explored by comparing genomic DNA sequences. By comparing the localization and number of exon–intron structures, 31 *ScHSF* genes were found to have different numbers of exons, ranging from 1 to 4. Moreover, five *ScHSF* genes had only one exon, while most *ScHSF* genes (20, ~ 64.5%) contained 2 exons, and all genes contained HSF-DNA binding sites (Additional file 3: Figure S2). In addition to this, *ScHSF1* and *ScHSF14* genes belonged to A2 subfamily, which have the same intron and exon structure with 4 exons and 3 introns, the highest number of *HSF* genes (Additional file 3: Figure S2a, b). It was worth mentioning that *ScHSF21* had a very large intron structure. In general, *ScHSF* genes of the same subfamily had similar gene structures. Subfamily A2 showed greater structural differences in the number of introns. Therefore, it could be speculated that they might have more biological functions.

To further evaluate the structural diversity of *ScHSF* genes, the motifs of *ScHSF* genes were analyzed using online motif software. Ten different motifs were identified in ScHSF proteins (motif 1 to motif 10). Motif 1, 2, 4, and 5 were usually located together, indicating that these four motifs were closely associated with SPL proteins. Notably, *ScHSF* genes of the same subfamily usually had similar motif compositions. For example, subfamily C contained motifs 1, 2, 3, 4, and 5, while subfamily A2 contained all the same motifs (motifs 1–9). Furthermore, it was found that some motifs were located at specific positions. For instance, motif 2 was always located at the beginning of the motif, while the motifs located at the end were different. Motif 5 was always between motif 1 and motif 2 (Additional file 3: Figure S2c). Overall, these results indicated that genes from the same subfamily had similar genetic composition and structure tended to cluster together, which was consistent with the population classification of the phylogenetic tree.

### Chromosomal spread and gene duplication in *ScHSF* genes

**Fig. 2:**
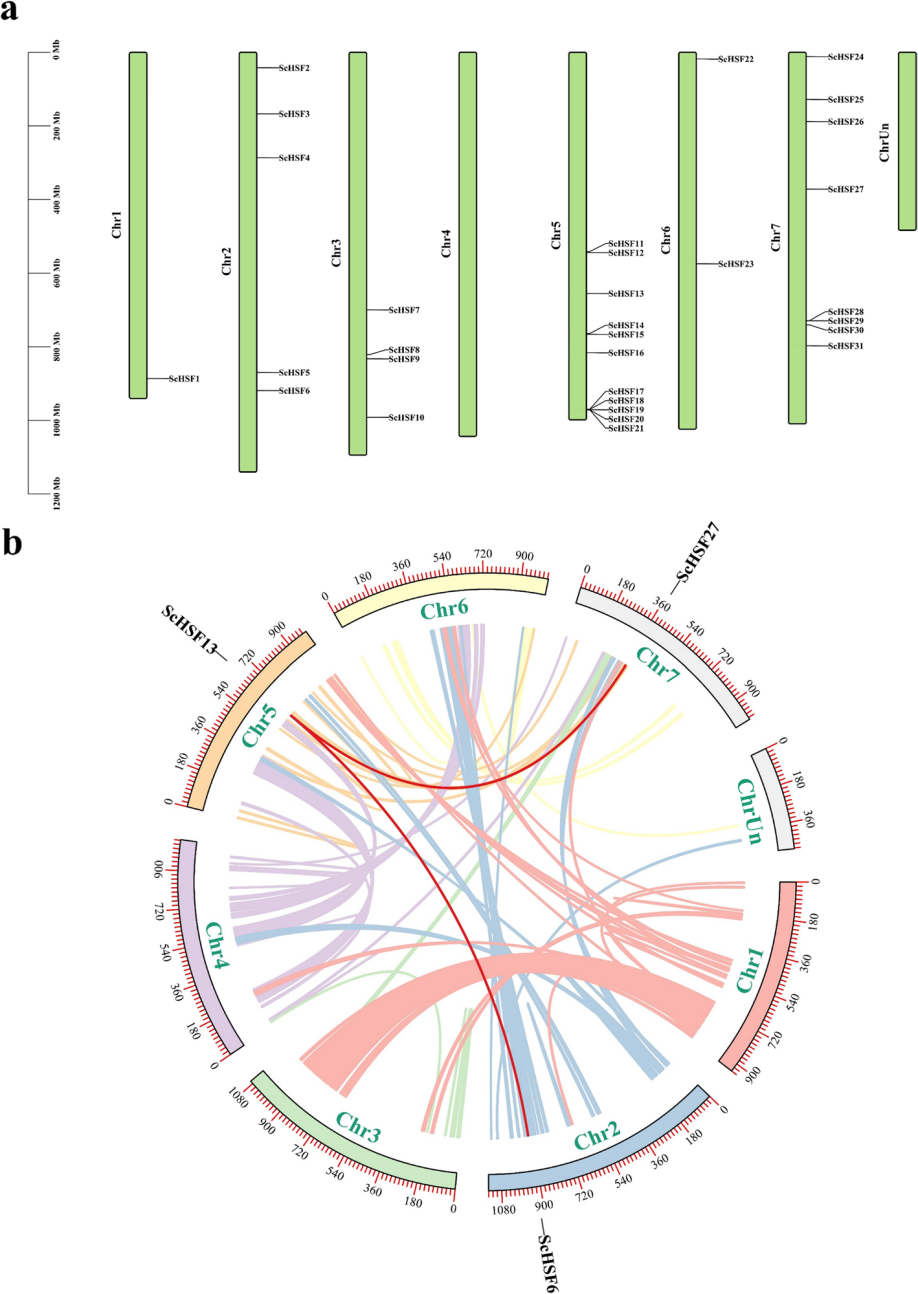
The chromosomal distribution and synteny blocks of HSF genes in *Rye*. **a** Distribution of the 31 *ScHSF* genes on different chromosomes. The scale represented the length of chromosomes, whereas the green bars indicated chromosomes. The chromosome number is displayed on the left side of each green bar. **b** Analysis of interchromosomal fragment duplication of *HSF* genes in the *Rye* genome. The colored lines represented all synthetic blocks and the red lines specifically indicated the duplicated pairs among the 31 *ScHSF* genes

According to the newly published *Rye* genome database, *HSF* genes (31) were distributed on seven chromosomes (Chr), and each *HSF* gene was named based on its physical position on chromosomes. Chr5 contained the most *ScHSF* genes (11, ~ 35.5%), followed by Chr7 (8, ~ 25.8%). Chr1 had only 1 gene (~ 4.76%), while Chr4 had no distribution of *ScHSF* genes. Notably, nearly half of *HSF* genes were located at the bottom of chromosomes (Fig. 2a).

Gene duplication events, including tandem repeat events and segmental duplications, play an essential role in gene amplification and the generation of new functions, [34]. Chromosomal regions with a range of 200 kb containing two or more genes are defined as tandem repeat events [35]. Accordingly, a duplication event analysis of *HSF* genes in *Rye* was performed to explore the evolutionary conservation of gene family. The results showed that there were no tandem duplication events in *Rye* genome, but two pairs of duplicated fragments were present (Fig. 2b, Additional file 4: Table S2). Four homologs in *HSF* genes indicated an evolutionary relationship between these genes. The highest number of *ScHSF* was found in LG5 (*n* = 2), followed by LG2 and LG7 (*n* = 1), and all genes were linked within subfamily B, suggesting that some *ScHSF* genes might be accompanied by fragment replications. These replication events were the main drivers of new functions of *ScHSF* genes during evolution.

### Evolutionary analysis of the *ScHSF* genes and *HSF* genes of several different species

**Fig. 3:**
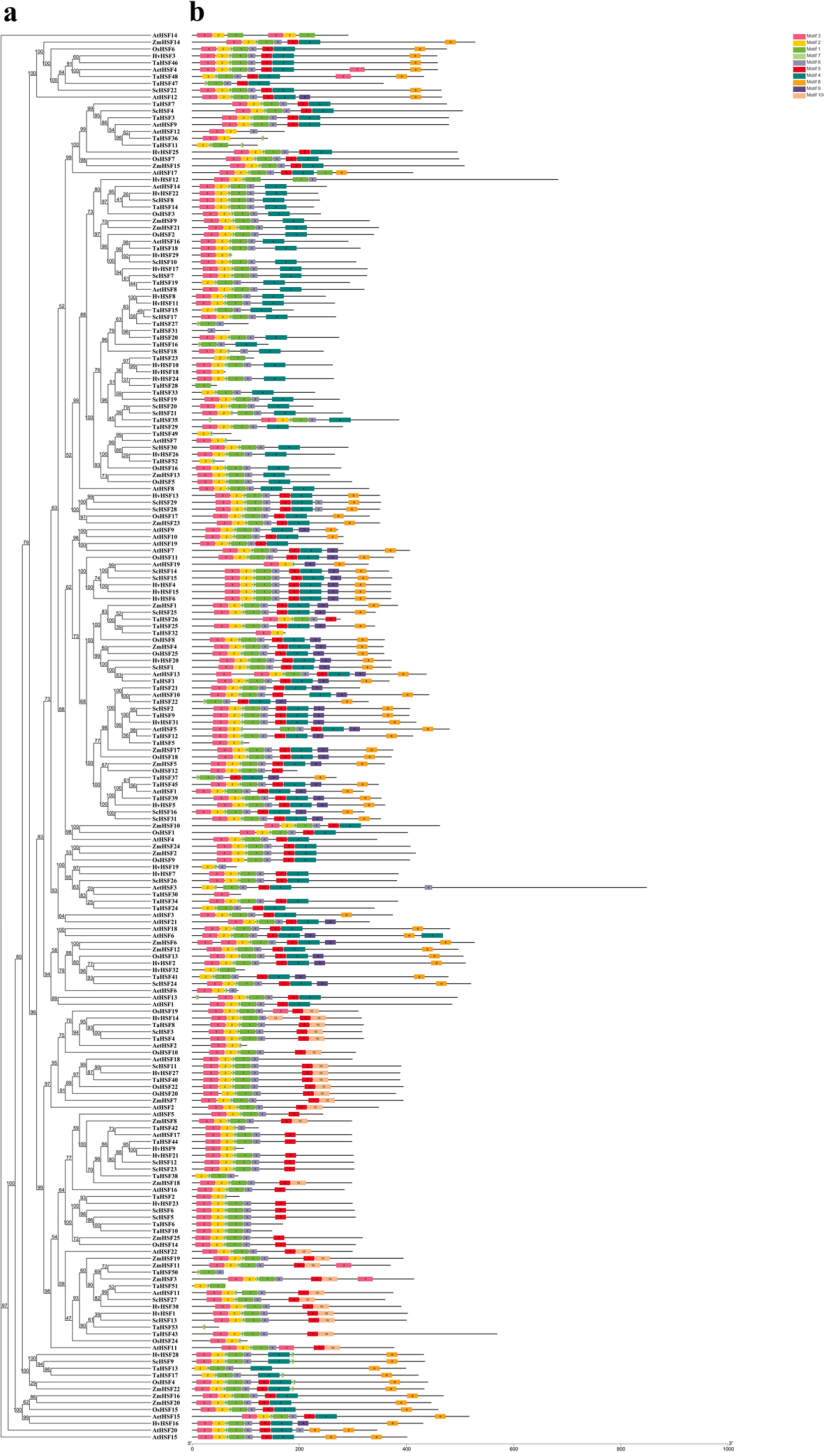
Phylogenetic relationships and motif compositions of HSF proteins of seven different plant species (*Rye*, *A. thaliana*, *H. vulgare*, *O. sativa*, *Z. mays*, *T. aestivum*, *and A. tauschii* Coss). **a** An unrooted phylogenetic tree was constructed using the neighbor-joining method as implemented by Geneious R11. **b** Distribution of the conserved motifs in HSF proteins. Ten differently colored boxes represent different motifs and their position in each HSF protein (Table S3)

A dicotyledon (*A. thaliana*) and five monocotyledons (*H. vulgare*, *O. sativa*, *Z. mays*, *T. aestivum* and *A. thuschii* Coss) were selected to analyze the evolution of *HSF* in *Rye*. 31of *ScHSF* genes identified were compared with 10 conserved motifs from 6 other plants. *ScHSF* genes were not evenly distributed in the phylogenetic tree. As shown in Fig. 3a, the ScHSF proteins tended to gather with the HSF proteins of *T. aestivum* and *H. vulgare*, suggesting that they are more closely related (Fig. 3a). These genes, from the same subfamily, tended to have the same themes and tended to cluster together. Remarkably, almost all *HSF* genes from these seven plants contained motifs 1, 2, 3, 6, and 7 (Fig. 3, Additional file 5: Table S3). Subfamily A2 contained the most motifs and showed a diversity of expression. In addition, all genes except *HSF* in wheat began with motif 3. And in subfamily A1, A2, A3, and C, motif 8 was always distributed at the end of the pattern. In summary, *ScHSF* genes in subfamily A3 had high homology with barley *HSF* gene clusters, while most of the HSF genes in other groups had homology with wheat *HSF* gene clusters, indicating a closer distance in evolution and with similar potential functions.

A homology map between *Rye* and six representative species was constructed to explore the phylogenetic mechanisms of *HSF* genes in *Rye*. These species included one dicot (*A. thaliana*) and five monocots (*H. vulgare*, *O. sativa*, *Z. mays*, *T. aestivum*, and *A. tauschii* Coss). A total of 31 *ScHSF* genes were colinear with those of *A. tauschii* Coss (19), *A. thaliana* (21), *Z. mays* (25) and *O. sativa* (26), *H. vulgare* (32), and *T. aestivum* (82). The number of homologous pairs among the other six species (*A. thaliana*, *H. vulgare*, *O. sativa*, *Z. mays*, *A. tauschii* Coss, and *T. aestivum*) was 1, 19, 19,21, 26, and 69, respectively (Fig. 3, Table S3).

**Fig. 4:**
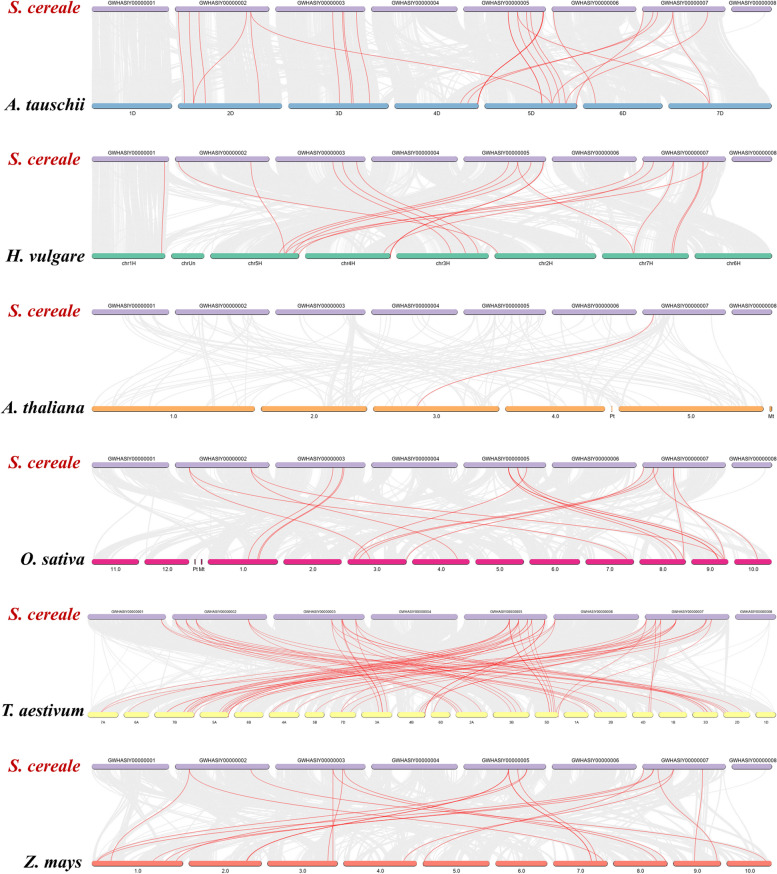
Analysis of the *HSF* genes between *rye* and six representative plant species (*A. thaliana, H. vulgare, O. sativa, Z. mays, T. aestivum, and A. tauschii* Coss). Gray lines in the background indicate the neighboring blocks in genomes of *rye* and other plants, whereas red lines highlight the syntenic rye *HSF* gene pairs

Homology analysis of these six plants revealed at least one pair of genes homologous to *ScHSF*, such as *ScHSF25* with *AET4Gv20678400.5/ Zm00001d032923_T002/ AT3G22830.1/ HORVU4Hr1G073650.2/ Os10t0419300-01/ TraesARI4B01G298300.1*, indicating that these homologous genes were highly conserved and might have existed prior to the ancestral divergence. Accordingly, it was speculated that they might have played a crucial role in the evolution of HSF gene family in *Rye*. Interestingly, In *H. vulgare*, *O. sativa*, *A. tauschii* Coss, *Z. mays*, and *T. aestivum*, gene pairs were found to be collinear with eight *ScHSF* genes (*ScHSF6*, *ScHSF7*, *ScHSF8*, *ScHSF12*, *ScHSF13*, *ScHSF24*, *ScHSF25*, and *ScHSF27*) (Fig. 4, Additional file 6: Table S4). These homologous gene pairs could have been formed by gene replication during the differentiation of dicotyledons and monocotyledons.

### Analysis of cis-acting elements in *ScHSF* promoters

**Fig. 5:**
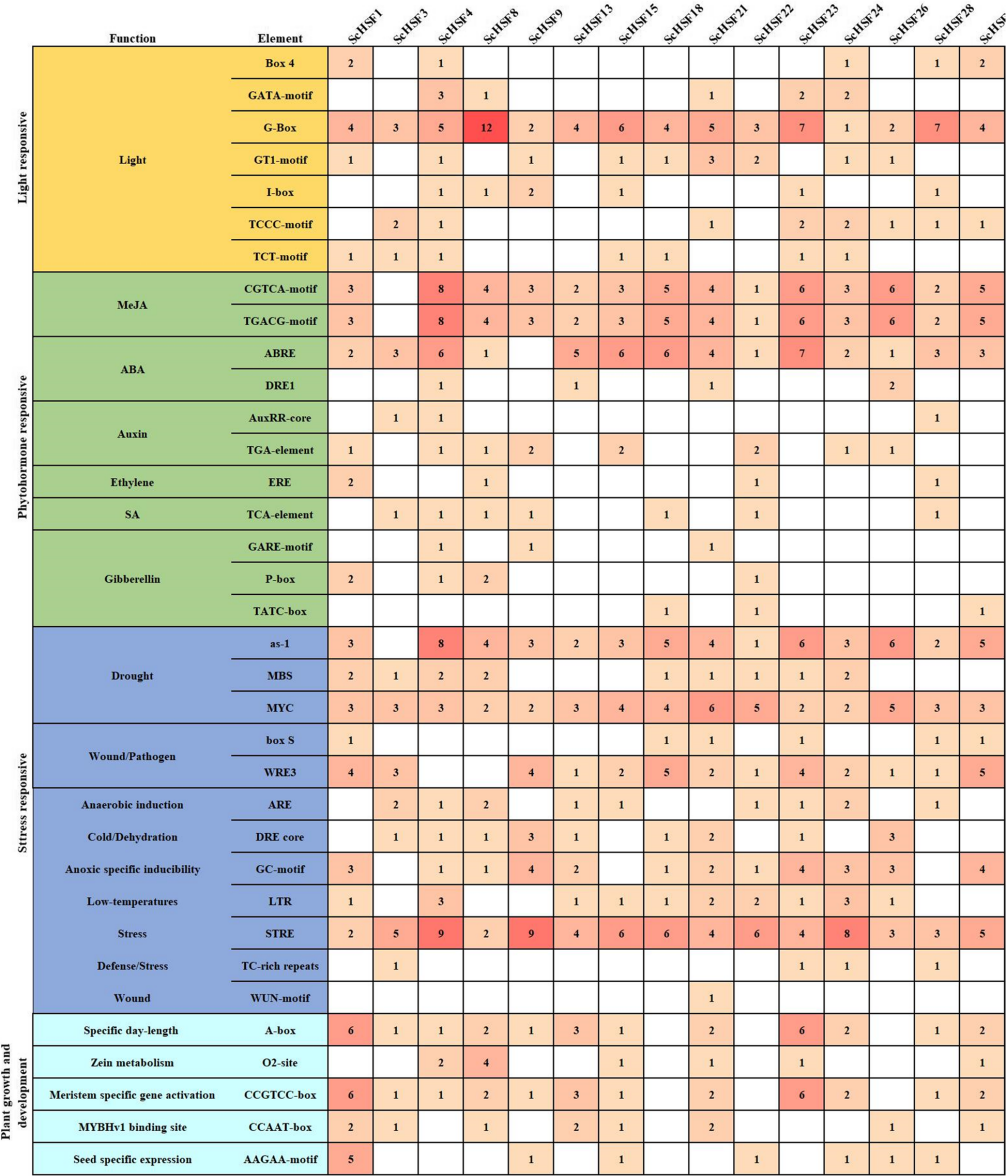
The distribution of cis-acting elements in promoters of *ScHSF* gene family members

The promoter regions of *ScHSFs* were analyzed to provide ideas for tissue-specific expression of genes and stress response patterns. The cis-acting elements in promoter could be divided into four categories: light-responsive, hormone- and stress-responsive, plant growth- and development-related elements. Individual *ScHSF* gene in *Rye* covered most of phytohormone response elements, including abscisic acid response elements (ABRE). MeJA hormone response elements (CGTCA-motif and TGACG-motif). In addition, cis-regulatory elements were associated with low temperature, drought, anaerobic conditions, and other defenses found in all *ScHSF* genes (Fig. 5, Additional file 7: Table S5).

All genes contained light (G-BOX), drought (MYC), and stress (STRE) elements, while 93.3% of *ScHSF* genes contained MeJA and ABA response elements. The promoters of *ScHSF1*, *ScHSF8*, *ScHSF22*, and *ScHSF26* contained growth hormone -, ethylene -, SA-, and gibberellin- reaction elements. All *ScHSF* promoters except the *ScHSF3* promoter contained drought-related element as-1 (Fig. 5, Additional file 7: Table S5). These results suggested that certain cis-acting elements might be involved in regulating the expression of different tissues, such as seeds and meristematic tissues. Furthermore, we speculated that *ScHSF* genes might be involved in tissue developmental processes and in response to various abiotic stresses.

### Protein–protein interaction network analysis of *ScHSF* family members

**Fig. 6:**
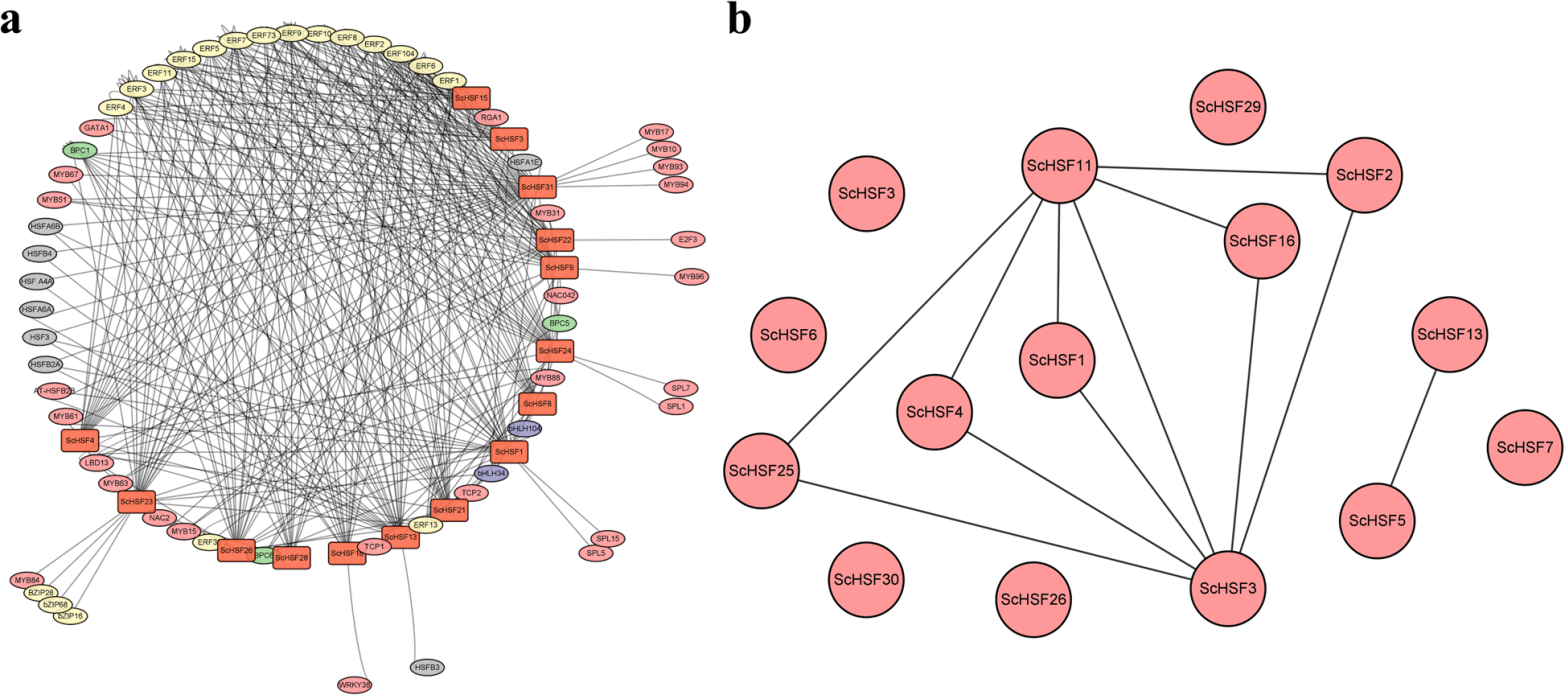
Predicted interactions between rye HSF proteins. **a** Regulation network between *ScHSFs* and potential TFs. Red boxes represent *ScHSFs* genes and different colored ovals represent different TFs. Yellow shows ERF, gray shows HSF, blue shows bZIP, green shows SPL, pink shows MYB. **b** Prediction of the protein– protein interaction network among 31 *ScHSFs*

The combination of promoter cis-elements and transcription factors can regulate the precise initiation and efficiency of transcription. The PlantTFDB was used to explore the potential TFs that binding to *ScHSF* promoter. The results showed that *ScHSF18* and *ScHSF15* had the most and least transcription factors, respectively (Fig. 6a). Meanwhile, All *ScHSF* genes were regulated by a large number of *ERF* transcription factors. Studies have shown that ERFs can regulate the expression of target genes JA-based target genes and defense against *Boea chinensis* in *Arabidopsis thaliana*, suggesting that *ScHSF* might indirectly participate in regulation of JA synthesis against pathogens. Meanwhile, it has been reported that *OsERF3* acts as a central switch that enables plant metabolism to respond appropriately to insects [36]. Therefore, it was speculated that *ScHSF* and participating in JA response played a potential role in insecticide traits through *ERF* regulation [37, 38].

To better understand regulatory relationships of *HSF* genes in *Rye*. We performed mutual protein prediction of HSF genes in *Rye* based on the most homologous wheat species. As shown in Fig. 6b, interactions existed among the nine *ScHSF* members. Moreover, *ScHSF5* and *ScHSF13* could interact with each other. Interestingly, *ScHSF3* and *ScHSF11* could interact with each other, along with *ScHSF1*, *ScHSF2*, *ScHSF4*, *ScHSF16*, and *ScHSF25*. Overexpression of *AtHSFA2* (homologous *ScHSF1*, *ScHSF2*, *ScHSF16*, and *ScHSF25*) significantly increased basal and acquired heat tolerance in *Arabidopsis* plants [39]. *ScHSF3*, *ScHSF5*, *ScHSF11*, and *ScHSF13*, were identified as homologs proteins of *Arabidopsis* HSFB2b, a protein involved in plant resistance to pathogens [40].

### Expression patterns of *ScHSF* genes in different plant organs

**Fig. 7:**
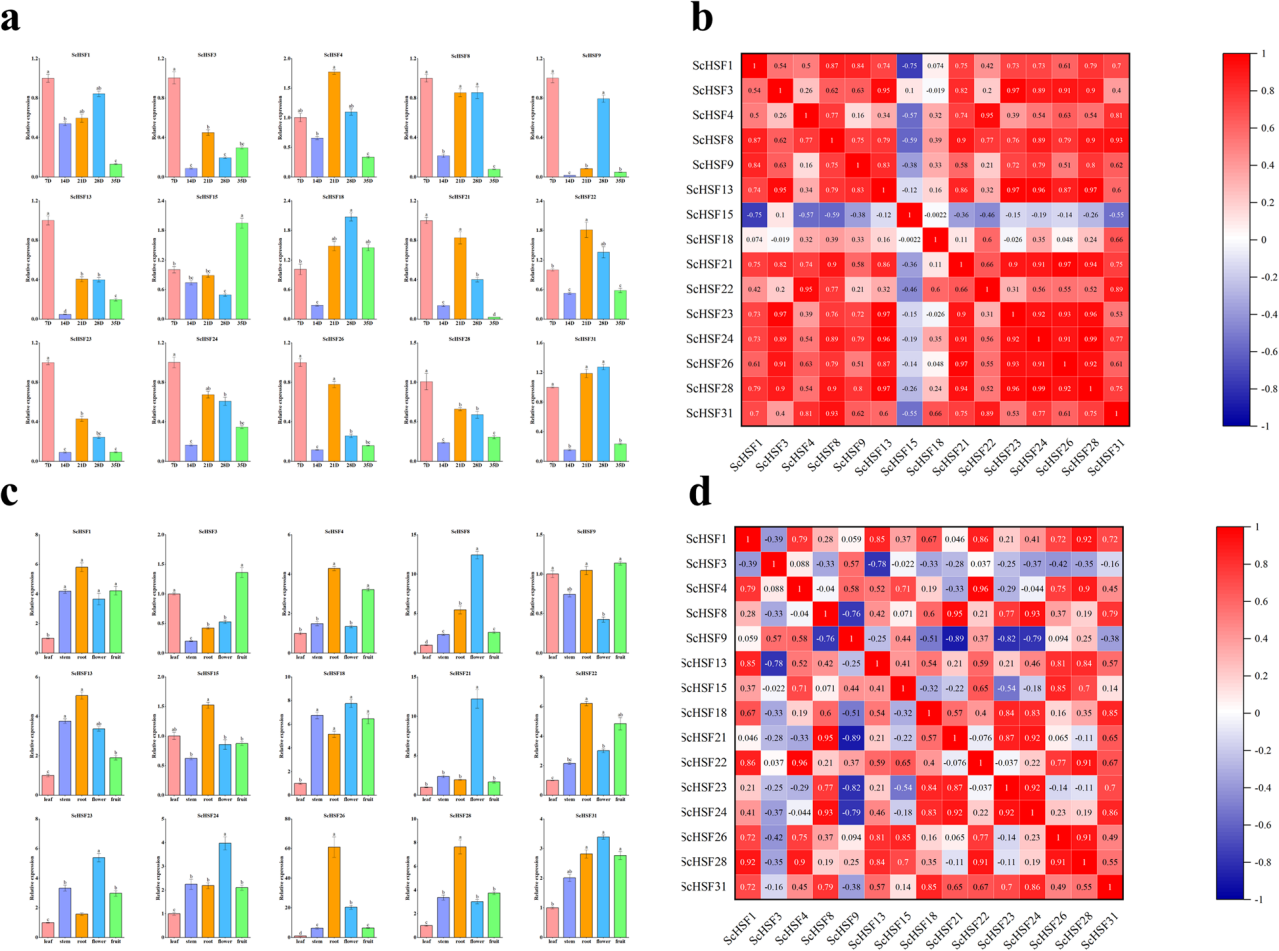
Tissue-specific gene expression of the 15 *ScHSF* genes and gene expression levels during various fruit development stages. **a** Expression patterns of the 15 *ScHSF* genes in flower, leaf, root, stem, and fruit tissues were analyzed using qRT-PCR. Error bars represent the stand errors with three replications. Lowercase letters indicate significant differences among treatments (α = 0.05, LSD). **b** Positive number = positive correlation; negative number = negative correlation. Red numbers indicate a significant correlation at the 0.05 level. **c** Expression patterns of the 15 *ScHSF* genes at different fruit developmental stages were analyzed using qRT-PCR (7 DPA, 14 DPA, 21 DPA, 28 DPA, and 35 DPA). Error bars represent the stand errors with three replications. Lowercase letters indicate significant differences among treatments (α = 0.05, LSD). **d** Positive number = positive correlation; negative number = negative correlation. Red numbers indicate a significant correlation at the 0.05 level

To further evaluate the potential function of *ScHSF* genes, a total of 15 genes in seven subfamilies were selected and the expression of these representative genes in four plant organs (root, stem, leaf, and flower) was analyzed by qRT-PCR. *ScHSF* genes showed different expression patterns in roots, stems, leaves, and flowers, suggesting that these genes contribute to diverse regulatory roles (*P* < *0.05*). All genes were expressed in different tissues; two genes (*ScHSF3*, and *ScHSF9*) had the highest expression level in fruits; seven genes (*ScHSF1*, *ScHSF4*, *ScHSF13*, *ScHSF15*, *ScHSF22*, *ScHSF26*, and *ScHSF28*) had the highest expression level in roots, while six genes (*ScHSF8*, *ScHSF18*, *ScHSF21*, *ScHSF23*, *ScHSF24*, and *ScHSF31*) had the highest expression level in flowers (Fig. 7a). Most genes from the same subfamily had similar expression patterns, suggesting that these genes might have similar functions. By analyzing the expression of *ScHSF* genes in different tissues, it was obvious that all *HSF* genes were least expressed in leaves. We could speculate that *HSF* genes might be relevant to the development of stems, roots, and flowers in plants (Fig. 7b).

The *ScHSFs* may also regulate the fruit development of *Rye*, thus affecting its nutritional composition and development rate [41, 42]. Therefore, 15 *HSF* genes were analyzed for expression level at five different post-anthesis stages (7d, 14d, 21d, 28d, 35d) after anthesis to identify genes that could potentially regulate *Rye* fruiting-related genes (*P* < *0.05*). Most *ScHSF* genes displayed different expression patterns at five stages of fruit development. In the fruit of *Rye*, the expression of five genes (*ScHSF4*, *ScHSF15*, *ScHSF18*, *ScHSF22*, and *ScHSF31*) were significantly increased with fruit development, while the expression of most genes (*ScHSF3*, *ScHSF13*, *ScHSF23*, *ScHSF26*, and *ScHSF28*) decreased with fruit development (Fig. 7c). The expression of most genes showed a down-regulation trend in fruit expression with increasing time, and it could be speculated that *HSF* genes showed negative regulation in fruits (*P* < *0.05*) (Fig. 7d). This also demonstrated that *HSF* genes played an essential role in fruit development and provided a theoretical basis for the studying of the nutritional value of *Rye*.

### Expression patterns of *ScHSF* genes under various treatments

**Fig. 8:**
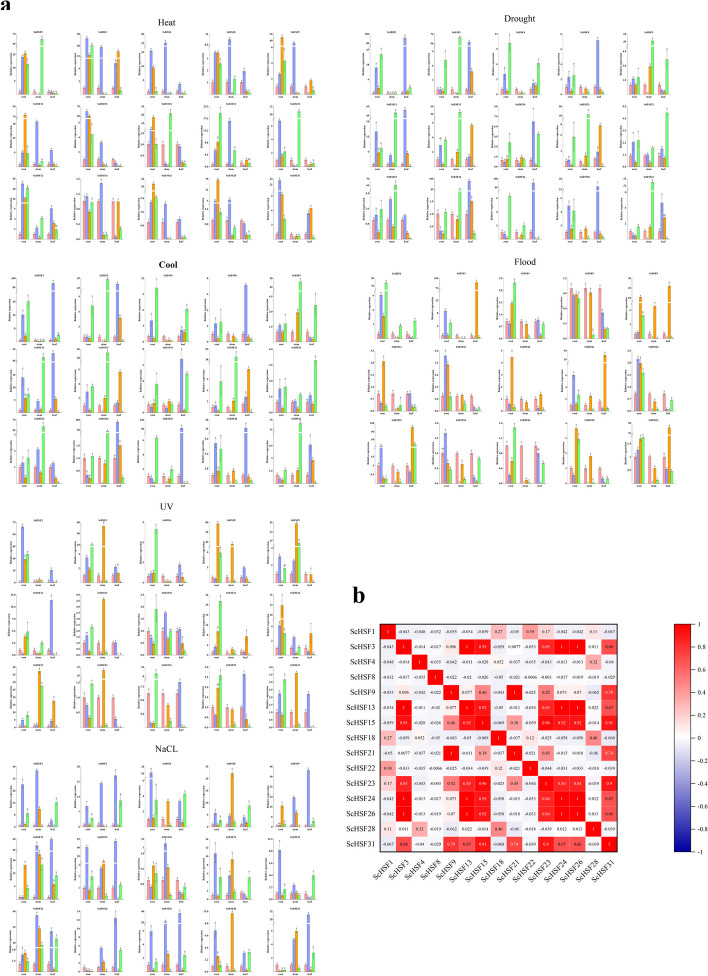
Expression analysis of the 15 *ScHSF* genes in three tissues (roots, stems, and leaves) at the seedling stage under different abiotic stresses (UV radiation, flooding, PEG, NaCl, heat, and cold treatments). **a** Expression analysis of the 15 *ScHSF* genes was performed using qRT-PCR. Error bars represent the stand errors with three replications and the lowercase letter above the bar indicates a significant difference (α = 0.05, LSD) among the treatments. **b** Positive numbers = positive correlations; negative numbers = negative correlations. Red numbers indicate a significant correlation at the 0.05 level

To determine whether the expression of *ScHSF* genes was restricted by different abiotic stresses, a representative expression of 15 *ScHSF* genes were expressed under six abiotic stresses. The results showed that some *ScHSF* genes exhibited significant up-regulated and down-regulated expression patterns under different stress treatments. Most of *ScHSF* genes also displayed significant differences in diverse tissues with the treatment period (*P* < *0.05*). For example, most *HSF* genes were induced by cold stress in stems and leaves, whereas most genes were induced to express in roots under heat stress. Notably, *ScHSF3*, *ScHSF9*, *ScHSF13*, *ScHSF15*, *ScHSF21*, *ScHSF23*, *ScHSF28*, and *ScHSF31* showed opposite patterns of highest expression levels in leaves and stems, compared to roots under cold and heat stress. Under flooding stress, expression level of *ScHSF1*, *ScHSF3*, and *ScHSF31* were most significantly up-regulated and mostly concentrated at 4 h treatment time. Under drought stress, *ScHSF1*, *ScHSF8*, *ScHSF18*, *ScHSF22*, *ScHSF24*, *ScHSF26*, and *ScHSF28* were significantly up-regulated in leaves. Meanwhile, most of the genes also showed a significant up-regulation under UV and NaCl stresses (Fig. 8). It should be emphasized that *ScHSF1* and *ScHSF3* were significantly highly expressed under all six different stress treatments and it could be further investigated as a potential candidate gene for stress management (*P* < *0.05*).

**Fig. 9:**
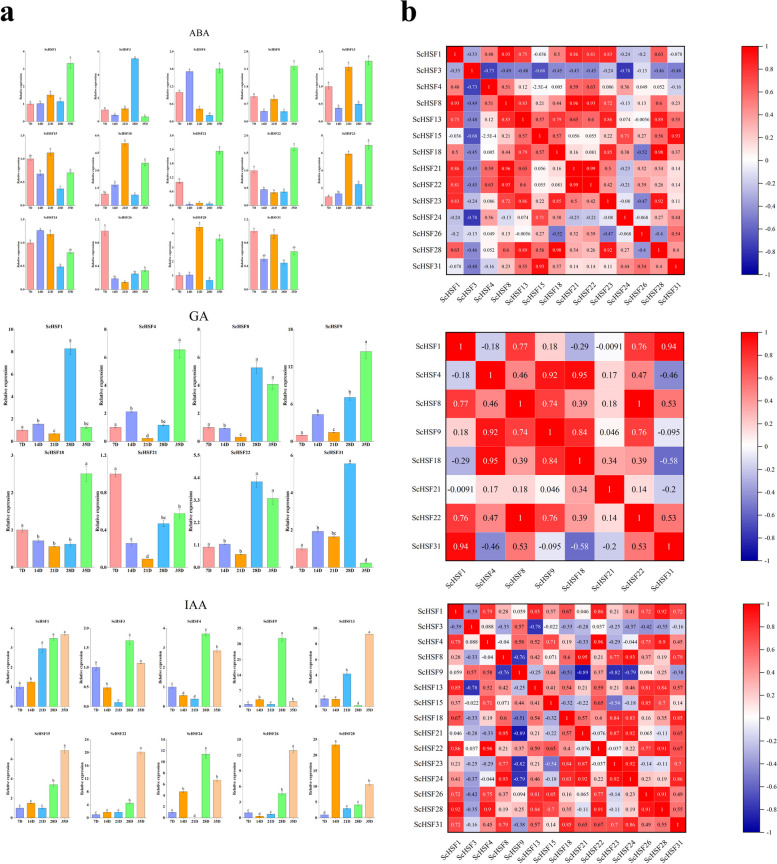
Expression analysis of the 15 *ScHSF* genes in fruits under different hormones (ABA, IAA, and GA3). **a** Expression analysis of the 15 *ScHSF* genes was performed using qRT-PCR. Error bars represent the stance error of three replicates, with lowercase letters above the error bars indicating significant differences among the treatments (α = 0.05, LSD). **b** Positive numbers = positive correlation; negative numbers = negative correlation. Red numbers indicate a significant correlation at the 0.05 level

In addition, the expression patterns of *ScHSF* genes under ABA, IAA, and GA3 were used to further explore the functions of genes. The genes exhibited different expression patterns under different hormone treatments (*P* < *0.05*) (Fig. 9). Under ABA treatment, most genes showed an up-regulation trend, while *ScHSF15*, *ScHSF24*, *ScHSF26*, and *ScHSF31* showed a down-regulation trend. Moreover, *ScHSF9* showed the highest expression level under IAA treatment, while only *ScHSF5* expression level was down-regulated. Expression level of *ScHSF22* and *ScHSF28* were the highest under IAA treatment.

## Discussion

### *ScHSF* gene structure and evolutionary analyses

During the growth and development of *Rye*, *HSF*, a transcription factor involved in various stress responses, including high-temperature stress, salt stress, drought stress, and oxidation stress, played a crucial role [31, 32]. In recent years, the rapid development of metagenomics has resulted in the identification and characterization of *HSF* genes in many plants, including *O. sativa*, *A. thaliana*, *Z. mays*, *Poplar*, *tomatoes*, *T. aestivum.* However, the study of *ScHSF* family was still poorly understood to date.

In this study, 31 *HSF* genes were identified in *Rye* with HSF proteins ranging from 260 to 520 amino acids in length (Additional file 1: Table S1). A comparative genomic analysis of gene structure revealed that all *HSF* genes contained different numbers of introns, ranging from 0 to 4. All of the encoded proteins showed complex and variable structures, while variability might be attributable to gene duplication during evolution. The introns, as a part of plant evolution, might not only increase the length of genes as well as the frequency of recombination between genes and played a major role in the regulatory roles [43]. In contrast, genes without introns have no advantages during the evolution of the species and delay regulatory responses [44–47]. Therefore, many *ScHSF* members respond rapidly when subjected to stress treatments. The same subfamily has a similar number of motif compositions and introns, which allows us to speculated that they might share a common evolutionary origin and molecular functions. This approach can also be used to predict the function of unknown proteins.

Based on the conserved structural domains of *Arabidopsis*, they were divided into seven subfamilies. Each group contains at least one *HSF* gene from *Arabidopsis* and *Rye*, suggesting that these genes have not been missing during evolution and might have some biologically important functions (Fig. 1a). Gene amplification is the main driver for the generation of new functional genes during evolution, which is divided into two types: segmental duplications and tandem replication. Compared to segmental replication [48], tandem duplication events reprensent a larger proportion of plant genomes, with an approximately 10% incidence in *Arabidopsis* and *rice* [49, 50]. We found more HSF proteins in *Rye* compared with *A. tauschii* Coss (19), *A. thaliana* (21), *Z. mays* (25), and *O. sativa* (26). This suggested a possibility of more gene duplication events happened in *Rye*, which could also lead to the production of new functional genes in plants to adapt to harsh environment [51]. Based on physical location, 31 *ScHSF* genes were unevenly distributed on 7 chromosomes of *Rye* (Fig. 2a). Homology analysis of the *HSF* gene in *Rye* showed that no tandem duplicate gene pairs were discovered. Nevertheless, two pairs of fragment duplicates were identified (Fig. 2b). The homologous genes on different chromosomes of *Rye* might have promoted the evolution of *ScHSF* genes, resulting in a higher number of *HSF* genes in *Rye* than in other monocotyledons (*A. tauschii Coss*, *Z. mays*, *and O. sativa*).

To further speculate on phylogenetic developmental mechanisms of *HSF* genes, six comparative syngeneic maps of *Rye* connections with one dicotyledon and five monocotyledons were constructed. As we can see from Fig. 3, *HSF* genes from both *Rye* and different plants were classified into seven taxa. *ScHSF* genes from subfamily A3 showed higher homology with barely *HSF* genes clusters, whereas most of *HSF* genes from other groups were clustered with wheat. Interestingly, there was at least one pair of co-linear genes between *ScHSF25* and *AET4Gv20678400.5/ Zm00001d032923_T002/ AT3G22830.1/ HORVU4Hr1G073650.2/ Os10t0419300-01/ TraesARI4B01G298300.1*, which might provide a theoretical basis for understanding whether they shared a common ancestor. Analysis of orthologous genes also illustrated that *ScHSFs* had the highest number of homologous gene pairs with wheat, indicating a higher level of homology among them. In addition, by analyzing the motif composition of *HSF* genes in plants, we found that *HSF* genes contained 10 motifs, with different subfamilies containing similar motifs. Moreover, *HSF* genes contained almost all motifs of the A2 subfamily. These results also reaffirmed that *HSF* genes in *Rye* were more closely related to wheat and might have a common ancestor.

### Expression patterns and function prediction of *ScHSFs*

The analysis of gene expression is often used as an essential step in providing useful clues for functional prediction [52]. In this study, the expression patterns of 15 genes, which were represented in seven subfamilies, were selected and explored in different tissues and at different developmental stages. The results showed that most of *HSF* genes were significantly expressed (more than a twofold difference). For instance, most genes were significantly up-regulated in stems and leaves under cold treatment, while all genes were significantly up-regulated under UV and drought treatment (Fig. 8). This explained the high adaptability of *Rye* crop in alpine or arid areas. Most of *HSF* genes were significantly up-regulated in response to stress in these six treatments, and the genes expression was mainly in leaves and stems. However, the expression of most of *HSF* were highest in roots, suggesting that roots played a key function under drought conditions. Notably, both *ScHSF1* and *ScHSF3* were expressed in response to all six stresses. It could be further validated as a potential candidate gene for improving crop breeding.

Previous studies have shown that *HSF* genes were mainly involved in several environmental stress responses, such as high-temperature, salt, drought, and oxidation stress [11, 31, 32]**.** ClassA *HSFs* are major regulators of heat stress and could induce the expression of resistance genes [53]. The high expression levels of most *HSF* genes under different stress treatments also suggested that *HSF* genes played a significant role in roots and leaves. For instance, Tahmina reports that overexpression of the *AtHSFB4* (*AT1G46264.1*) gene in *Arabidopsis* resulted in a shortened root length [28]. *ScHSF23*, the corresponding homologous gene, was up-regulated in roots for stress-responsive expressions under heat treatment and drought conditions. Based on the fact that homologous genes with similar structures may have similar functions, it is speculated that *ScHSF23* may be related to root growth and development. Overexpression of *AtHSFA2* in *A. thaliana* increases stress tolerance, and enhanced callus growth [49, 54]. The *ScHSF1*, *ScHSF 2*, *ScHSF 14*, *ScHSF 15*, *ScHSF 16*, *ScHSF 25*, *and ScHSF 31* belong to A2 subfamily, which had high similarity to *AtHSFA2*.. Meanwhile, we found that A2 subfamily had the most abundant motifs, *ScHSF1* and *ScHSF15* also showed significant up-regulation in stress treatment (Fig. 8). Finally, two genes from each subfamily were screened for qRT-PCR analysis and verification of functional traits. The results showed that these genes were significantly up-regulated in different tissues during stress treatment, suggesting that these genes might respond to stress through different tissues. Interestingly, these up-regulated genes were not only expressed in roots but also dominantly in leaves and stems (Fig. 8). Thus, we speculated that this was likely due to complex protein interactions that coordinated the expression of multiple genes through a network of feedback mechanisms [55].

## Conclusion

Altogether, identification and systematic analysis of *HSF* genes in *Rye* showed that the 31 *ScHSF* genes were unevenly distributed on 7 chromosomes that were classified into seven subfamilies. By comprehensively analyzing gene structures and conserved motifs of 31 putative *ScHSF* genes, we found that motifs and gene structures of the same family were similar and might have the same biological functions. Furthermore, fragments and tandem repeats were the main drivers of novel functions in *ScHSF* family. Fragment repeats might have more substantial contributions to the evolution of *Rye HSF* genes. Overall, we performed a preliminary analysis of the structure of *HSF* gene family in *Rye* and further detailed its expression pattern. The results indicated that *ScHSF* gene family played a critical role not only in stem and flower development but also in hormonal and abiotic stress response during *Rye* development.

## Materials and methods

### Gene identification

The whole *Rye* genome was downloaded from the Ensembl website (http://ensemblgenomes.org). *HSF* gene family members were obtained based on two BLASTp approaches (PFAM and SMART) [56–58]. Firstly, all possible HSF proteins were identified using BLASTp (score value ≥ 100, *e* value ≤ 1e-10) with reference to the trihelix protein sequence of *Arabidopsis*. Secondly, the PFAM protein family database (http://pfam.sanger.ac.uk) was used to produce a Hidden Markov Model (HMM)with HSF domains, and then an HMM model cutoff value of 0.01 in HMMER 3.0 was applied to compare HSF protein sequences of *Rye* (http://plants.ensembl.org/hmmer/index.html). The availability of the HSF core sequence was confirmed using PFAM and the SMART program (http://smart.emblheidelberg.de). A total of thirty-one *HSF* genes were identified and then used as initial sequences to confirm HSF proteins (https://blast.ncbi.nlm.nih.gov/Blast.cgi?PROGRAM=blastp&PAGE_TYPE=BlastSear-ch&LINK_LOC=blasthome) with BLASTp. Finally, several characteristics of the *HSF* genes, such as the sequence length, isoelectric point (pI), molecular weight (MW), and subcellular localization, were identified using ExPasy. A 2000 bp sequence upstream of the start codon (ATG) of the *ScHSF* gene was extracted from *Rye* genome using TBtools, followed by an analysis of the cis-acting elements using PlantCare (http://bioinformatics.psb.ugent.be/webtools/plantcare/html). Finally, TFs were predicted through PlantTFDB and shown by using Cytoscape [59, 60].

### *HSF* gene structure

Multiple protein sequence alignments based on the domain sequences in characterized HSF proteins of *A. thaliana* were created by ClustalW with default settings. The deduced amino acid sequences of HSF domains of different subfamilies were manually regulated using GeneDoc software and Mega 7.0. Furthermore, Gene Structure DiHSFay Server (http://gsds.cbi.pku.edu.cn) online program was applied to analyze the exon–intron structure of *HSF* genes. MEME Online Applications (http://meme.nbcr.net/meme/intro.html) were then employed to identify the protein sequences by adjusting the optimum motif width to 6 ~ 200 and the maximum number of motifs to 10.

### Chromosomal distribution and gene duplication events

All *ScHSF* genes were mapped to locations on different *Rye* chromosomes by using physical location information and handled using the Circos program [61]. The multiple collinear scanning toolkits (MCScanX) were then used, with default parameters, to analyze replication events of *ScHSF* genes [62]. Finally, the *HSF* genes homology between *Rye* and six other plants (*O. sativa*, *Z. mays*, *H. vulgare*, *A. thaliana*, *T. aestivum*, and *A. tauschii* Coss) was measured using Dual Synteny Plotter (https://github.com/CJ-Chen/TBtools).

### Phylogenetic analysis and classification of the ScHSF family

With regard to the classification of AtHSFs, all identified ScHSF proteins were first clustered into diverse groups. Next, a neighbor-joining (NJ) tree was built using Jukes-Cantor model in MEGA 7.0 [63]. The phylogenetic tree was then generated, with a bootstrap value of 1000, and was assigned by gene R11 and BLOSUM62 cost matrix. Moreover, we generated a multi-species phylogenetic evolutionary tree that included all HSF protein sequences from *Rye* as well as six others plants species (*O. sativa*, *Z. mays*, *H. vulgare*, *A. thaliana*, *T. aestivum*, and *A. tauschii* Coss). Notably, all protein sequences were downloaded from the UniProt database (https://www.uniprot.org). A protein–protein interaction analysis was performed on the STRING database (http://string-db.org) using ScHSFs as the queries and *T. aestivum* proteins as references. Promoter cis-acting elements were predicted by both PlantCare and PlantTFDB [60, 64].

### Plant materials, growth conditions, and different abiotic stress in *Rye*

The rye seed used in the experiment was provided by Fan Yu from Guizhou University. Wei ning Rye is the variety we used. Rye plants were cultivated in pots containing a mixture of soil and vermiculite (1:1) in a growth room. The growth room was maintained at a temperature regime of 25 °C during the 16-h daytime period and 20 °C during the 8-h nighttime period. The 3arelative humidity in the growth room was set at 75%. After 21 days of growth, stress treatment was initiated. Fruit sampling was conducted when the first seed setting occurred, and subsequent samples were collected every other week for five consecutive harvests.After planting, leaves, roots, stems, grains, anthers, and styles were collected from five individual plants under the same growth environment. The samples were immediately stored in liquid nitrogen at -80℃ until further analysis. The expression pattern of 31 *HSF* genes under different stresses was explored. Specifically, the abiotic stress treatments, including salt treatment (5% sodium chloride), water immersion (full plant), drought treatment (30% PEG 6000), UV radiation (70W/cm2, 220 V, 30W), high temperature treatment (40℃), and low temperature treatment (4℃), were applied at the seedling stage (after 21 days). Each stress treatment was replicated five times, and qRT-PCR analysis was performed after sampling at 1 h, 4 h, and 12 h, respectively. Hormone stress treatments were applied at seedling stage (after 21 days) with package expansions of ABA (100 μmol/L), IAA (100 μmol/L), and GA3 (100 μmol/L). Grain samples were collected at 7D, 14D, 21D, 28D, and 35D, and each treatment was replicated five times, respectively.

### Total RNA extraction, cDNA reverse transcription, and qRT-PCR analysis

Total RNA was extracted using a plant RNA extraction kit (Vazyme Biotech) following the manufacturer's instructions. A cDNA library was constructed through reverse transcription of 1 mg RNA samples using 5 × HiScript® Reverse Transcriptase (vazymes) and 4 × gDNA (vazymes) kits in accordance with the manufacturer's protocol. The expression of some representative genes was then analyzed by qRT-PCR, with at least three biological replicates. The primers used were designed by Beacon Designer 7 (Additional file 8: Table S6). Relative mRNA expression was normalized to the actin gene (GADPH) mRNA expression as internal control and was calculated using the delta-delta Ct (2^−ΔΔCt^) method [65].

### Statistical analyses

JMP6.0 (SAS Institute) was used to perform analysis of variance (ANOVA) tests; multiple comparison tests of ANOVA results were performed using the least significant difference (LSD) method at two difference significance levels *p* < 0.05* and *p* < 0.01**. Finally, histograms were generated using Origin version 8.0.

### Supplementary Information

**Additional file 1.**

**Additional file 2.**

**Additional file 3.**

**Additional file 4.**

**Additional file 5.**

**Additional file 6.**

**Additional file 7.**

**Additional file 8.**

**Additional file 9.**

## Data Availability

The whole genome sequence information of *rye* was obtained from the Ensembl genome website (http://ensemblgenomes.org/). In the experiment, the *rye* material used was provided by Yu Fan from Guizhou University. The datasets supporting the conclusions of this study are included in the article and its additional files.

## Abbreviations

*HSF*: Heat stress transcription factors
*ScHSF*: Secale cereale L. HSF
*q*RT-PCR: Quantitative real-time polymerase chain reaction
*AtHSF*: *Arabidopsis thaliana* *HSF*
HMM: Hidden Markov Model
pI: Isoelectric point
LG: Linkage group
DPA: Days post anthesis
