# Supplementary material for: Genome-wide identification, phylogenetic and expression pattern analysis of HSF family genes in the Rye (Secale cereale L.)
Source: BMC Plant Biol. 2023 Sep 20;23:441. doi: 10.1186/s12870-023-04418-1 (PMC10510194; doi:10.1186/s12870-023-04418-1)
Supplement: Supplementary file 2 — Additional file 2. [file 12870_2023_4418_MOESM2_ESM.docx]

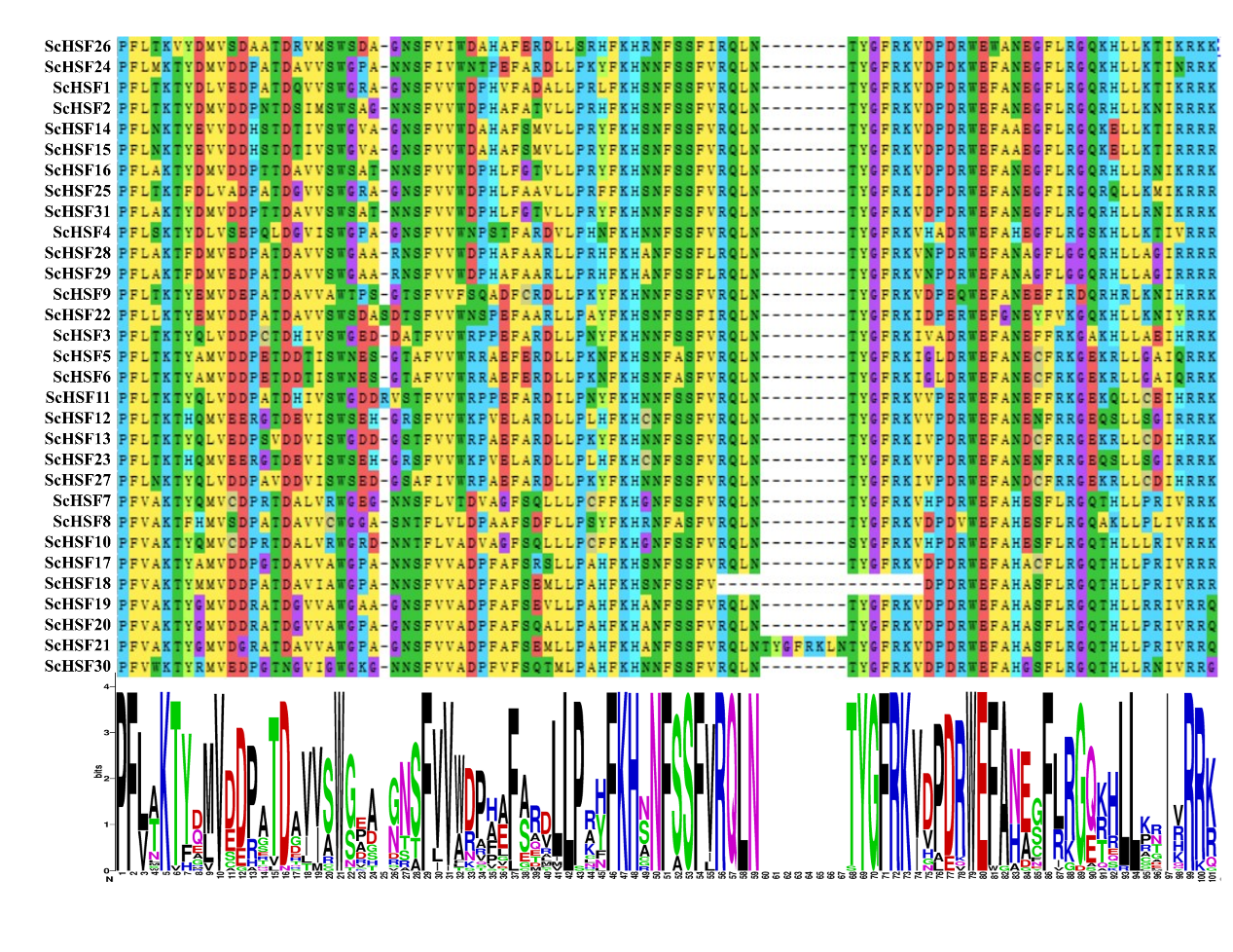


**Additional file 2 Figure S1:** Alignment of multiple ScHSF and select DBD domain amino acid sequences
